# The Italian paediatric society raccomandations on children and adolescents extra-domestic activities during the SARS COV-2 emergency phase 2

**DOI:** 10.1186/s13052-020-00826-3

**Published:** 2020-05-19

**Authors:** Alberto Villani, Elena Bozzola, Paolo Siani, Giovanni Corsello

**Affiliations:** The Italian Pediatric Society, Rome, Italy

**Keywords:** Coronavirus infection, Quarantine, Children, Outdoor activities, Social distancing

## Abstract

**Background:**

Due to novel coronavirus infection emergency, restricting measures have been imposed in Italy. As well as adults, also children are limited in their daily routine.

**Main text:**

As the lockdown period is expected to end on 3rd May 2020, we discuss the opportunity for children to practice safely recreational or physical activity outdoor.

**Conclusion:**

The Italian Paediatric Society recommends specific recreational and physical activities according to the age of the children and respecting social distancing.

## Background

In these months, Italian government imposed a national quarantine, restricting the movement of the population except for necessity, work, and health circumstances, in response to the growing pandemic of novel coronavirus infection (COVID-19) in the country, responsible of severe acute respiratory syndrome coronavirus 2 (SARS-COV2) disease. The restrictive measures also involved the paediatric population (0–18 year old subjects), which accounts for almost 10,000,000 citizens. In fact, the schools have been closed and the recreational activities outside home banned.

The Italian government continues to consult its panel of scientific experts on when and how to start the so-called “phase two”, an intermediary period between the current strict lockdown and “phase three”, during which the country will begin its gradual return to normality.

Schools are expected to remain closed until at least September. From September, ministers are considering a staggered or partial reopening of schools, or perhaps having all classes taught remotely. Other restrictions are expected to stay in place for many months.

Nevertheless, it is important to plan strategies in order to allow the paediatric population to leave their house safely. To the best of our knowledge, symptoms in children are generally absent or range from mild to moderate. The course is rarely serious, requiring intensive care [1, 2]. Two months after the first confirmed case of COVID19 in Italy, only two COVID 19 correlated deaths were reported in children affected by pre-existing severe comorbidities.

Children requiring high medical assistance and care needs, such as those with neuropsychiatric symptoms, important disabilities, or chronic disease, are about 1,000,000 individuals.

The lockdown period is due to expire on May 3. After a 2 month national quarantine**,** it should be important to leave the house as soon as possible, hopefully just from 4 May 2020.

Moreover, it is important to plan strategies to let either children or adults to practice safely recreational and physical activity outdoor. Consequentially, the modality (with or without a personal protective face mask), the period time (according to age and mental status), the setting (such as public gardens, playground, etc) should be defined.

Concerns are related to the unavailability of either published studies and specific face masks age sized.

## Recommendations

The Italian Paediatric Society recommends specific recreational and physical activities according to the age of the children and respecting social distancing.

According to the Centers for Disease Control and Prevention, social distancing or “physical distancing,” means keeping space between two people. To practice social or physical distancing, one person is supposed to:

- stay at least 2 m from other people
- not gather in groups
- stay out of crowded places and avoid mass gatherings (https://www.cdc.gov).

In details The Italian Paediatric Society agree on allow children outside but under strict conditions:

- infants aged from 0 to 17 months and still not able to walk can go out for a walk using a stroller. Respect social distancing also in outdoor spaces among companions.
- infants aged from 18 to 36 months can go out for a walk using a stroller or on their foot. Respect social distancing also in outdoor spaces among companions. If able to walk, they should be accompanied by an adult (aged over 18 years) respecting social distancing among either companions or children.
- children aged 37 months-6 years, who generally like gathering in groups, are difficult to be supervised. They can go outside, respecting social distancing also in outdoor spaces among companions and other children.
- children aged 7–13 years generally respect rules. They can go outside, accompanied by an adult, respecting social distancing also in outdoor spaces.
- children aged 14–18 years can go outside, respecting social distancing also in outdoor spaces and practice activities such as walking, bicycling, running.
- children aged 0–18 years affected by neuropsychiatric or chronic diseases can go outside, accompanied by an adult, respecting social distancing also in outdoor spaces.

Activities should be performed individually, in the proximity of personal residence, choosing the closest setting such as public garden, playground, park and maintaining interpersonal distance. Except for children affected by neuropsychiatric or chronic diseases, public means of transport should be avoid and personal means of transportation reduced. Moving on foot should be encouraged.

Of note, facial masks should be realized, according to the age of the children. Wearing cloth face coverings in public settings, such as schools, where other social distancing measures are difficult to maintain are important (https://www.cdc.gov).

In details, the Italian Paediatric Society suggests:

- in case of 0–36 months year old children, at nursery, personal protective equipment should be used by caregivers
- in case of 37 months to 6 years old children facial masks should be elastic to fit children face and made by hypoallergenic and breathable material to avoid suffocation.
- children aged 7–13 years may use adult facial masks
- adolescentes aged 8-14  years may use adult facial masks
- in case of children affected by neuropsychiatric or chronic diseases, caregivers should use facial masks. In children over 36 months, a size matched FP2 mask is suggested.

Facial masks used by both children and their caregivers should be the most protective available equipment.

## Conclusion

The Italian Paediatric Society suggest that children can enjoy the outdoors and being active, but under strict conditions according to their age and mental status.

## Data Availability

At the Italian Paediatric Society Secretary Office.

## Abbreviations

COVID-19: Novel coronavirus
SARS COV 2: Severe acute respiratory syndrome coronavirus 2
